# A Three-Ratio Scheme for the Measurement of Isotopic Ratios of Silicon

**DOI:** 10.6028/jres.098.017

**Published:** 1993

**Authors:** Harry Ku, Frank Schaefer, Staf Valkiers, Paul De Bièvre

**Affiliations:** National Institute of Standards and Technology, Gaithersburg, MD 20899-0001; Institute for Reference Materials and Measurements, Commission of the European Communities, JRC, B-2440 Geel, Belgium

**Keywords:** atomic weight of silicon, isotope ratios, mass spectrometer, measurement scheme, redundancy, symmetry

## Abstract

This paper proposes a scheme of measurement sequences that has been used for the redetermination of the molar mass (atomic weight) of silicon at the Central Bureau for Nuclear Measurements (now Institute for Reference Materials and Measurements). This scheme avoids correlations among the measured ratios caused by normalizing all ion current measurements to that of the largest ion current. It also provides additional information for checking on the consistency of these ratios within a cycle of scans. Measurements of isotope abundance ratios of silicon are used as an illustration.

## 1. Measurement Scheme

In using mass spectrometers to measure abundance ratios of isotopes in an element, a general procedure is to relate all minor abundant isotopes to one major abundant isotope at the same point in time. Ion currents in a scan of the isotopic ion beams are either adjusted for time differences or normalized by a symmetrical arrangement of measurement sequence followed by averaging.

This paper proposes a scheme of measurement sequences that has been used for the redetermination of the molar mass (atomic weight) of silicon at the Central Bureau for Nuclear Measurements (CBNM).^1^ This scheme avoids correlations among the measured ratios caused by normalizing all ion current measurements to that of the largest ion current. It also provides additional information for checking on the consistency of these ratios within a cycle of scans. Measurements of isotope abundance ratios of silicon are used below as an illustration.

Recalling that there are three stable isotopes of mass numbers 28, 29, and 30, with atomic masses *M*(^28^Si), *M*(^29^Si), and *M*(^30^Si) known almost exactly [3], the molar mass of a sample of silicon, *M*, can be determined as:

$$M\left(\mathrm{Si}\right)=\Sigma \;M\left({\;}^{i}\mathrm{Si}\right)f_{i}$$

where *f_i_* is the fractional isotope abundance of isotope ^*i*^Si, with *∑f_i_* =1.

In our experiment, measurements were made on SiF_4_ gas samples by means of a gas mass spectrometer equipped with a Faraday-cup collector. Ion currents were measured for ^28^SiF_3_^+^ (mass position 85), ^29^SiF_3_^+^ (86), and ^30^SiF_3_^+^ (87). Ion current ratios, *R*(*i/j*), are formed from these measurements. These ratios, after corrections by proportionality factors, are estimates of ratios of fractional abundances, *f_i_/f_j_*. Denoting the corrected ratios as *r*(*i*/*j*) and equating these to the *f_i_/f_j_*, it follows that

$$f_{28}=\frac{1}{1+r\left(29/28\right)+r\left(30/28\right)},$$ (1)

and

$$\begin{array}{l} f_{29}=r\left(29/28\right)f_{28},\\ f_{30}=r\left(30/28\right)f_{28}. \end{array}$$

Scaling the conversion of ion-current ratios into isotope-abundance ratios, i.e., determining the proportionality factors is accomplished by means of accurately synthesized isotope mixtures with isotope abundance ratios close to those of the unknown silicon samples.

**Fig. 1:**
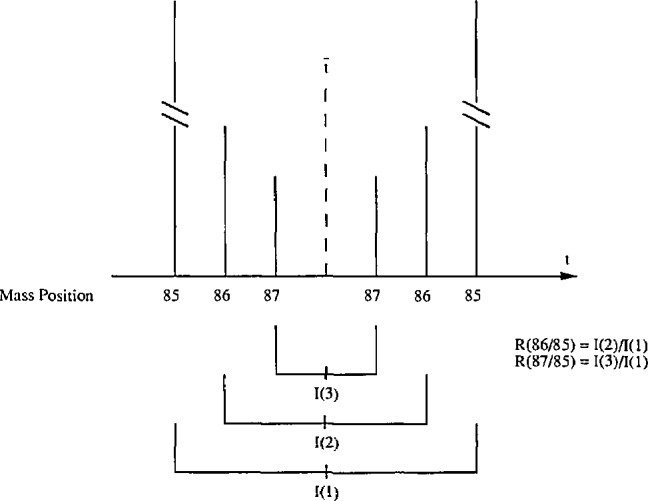
Two ratio scheme. One scan of a two ratio scheme is shown here where two measurements of ion currents of eaeh isotopic ion beam are made, centered about $\bar{t}$. Averages of those three pairs of measurements, *I*(1), *I*(2), and *I*(3), are used to form the two ratios *R* (86/85) and *R* (87/85).

In previous work [1], a two-ratio scheme was used, because Eq. (1) requires only the measured values of *r*(29/28) and r(30/28), i.e., the ratios of minor abundant isotopes (3^+^ to 4^+^%) to the major abundant isotope ^28^Si (92^+^%) The sequence of measurements in a scan proceeds as depicted in Fig. 1. There is one average for each intensity measured: *I*(1) for ^28^SiF_3_^+^, *I*(2) for ^29^SiF_3_^+^, and *I*(3) for ^30^SiF_3_^+^ Thus the ratios are:

R(86/85)=I(2)/I(1);R(87/85)=I(3)/I(1)

Eight to ten scans are measured during one cycle. A linear least squares fit of these measured ratios vs time would yield estimates of the ratios at *t*_0_, the instant sample gas is introduced into the ionizing chamber, to calculate and to allow for mass fractionation effect. The residual standard deviation, and the relative standard deviations of the *R*’s are also computed for each cycle.

The two-ratio scheme has been in use and appears to work well. However, two questions remain unanswered:

1. Since both *R* (86/85) and *R* (87/85) used the same value of *I*(85) in the denominator, the two ratios are correlated. What is the effect of correlation on the resulting calculated *f*_28_, *f*_29_, and *f*_30_? In addition, if the intensity of the major isotope is measured higher (or lower) than it should, because of minor nonlinearity in the instrumentation system, then the corresponding abundance will also be higher (or lower).
2. How do we know if the intensities measured in a scan, or in a cycle, are correct or consistent?

**Fig. 2:**
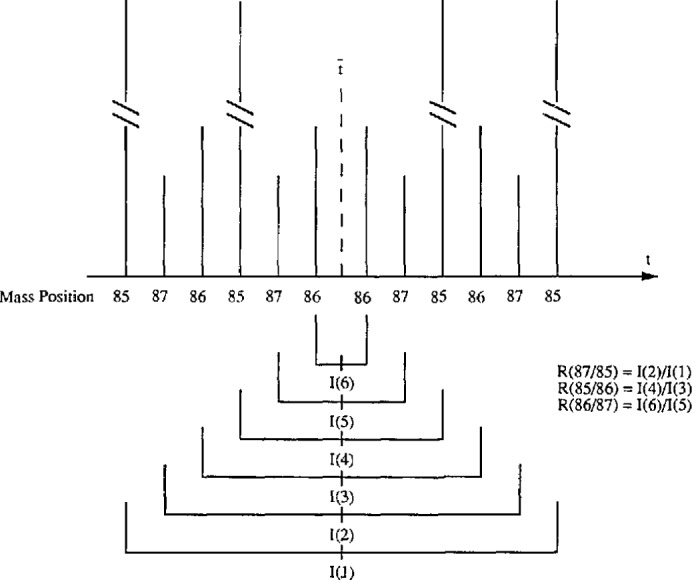
Three ratio scheme. One scan of a three ratio scheme is shown in this diagram where four measurements of ion currents of each ion beam are made, all centered about $\bar{t}$. Averages of each two measurements equidistant about $\bar{t}$, *I*(1) through *I*(6), are used to form the three ratios *R* (87/85), *R* (85/86) and *R* (86/87).

To answer these questions, a three-ratio scheme is devised such that all three ratios, *R* (87/85), *R* (85/86), and *R* (86/87), are measured, using six independently measured averages of intensities. Thus 12 ion intensities are measured in a sequence in one scan as shown in Fig. 2. For the scheme shown, there are two average intensities for 85, *I*(1) and *I*(4), two averages for 86, *I*(3), and *I*(6), and two averages for 87, *I*(2), and *I*(5). The ratios may be formed as

$$\begin{array}{c} R\left(87/85\right)=I\left(2\right)/I\left(1\right),\;R\left(85/86\right)=I\left(4\right)/I\left(3\right),\;\text{and}\\ R\left(86/87\right)=I\left(6\right)/I\left(5\right). \end{array}$$

Hence, there is no obvious correlation among the ratios to worry about because all ratios are formed with independently measured intensities. Furthermore, the “redundant” third ratio *R* (86/87) can be used to form a “closure” to check on the consistency of the measured ratios [4], i.e.,

R(87/85)R(85/86)R(86/87)≡1,

if there are no measurement errors. With measurement errors, we may formulate

R(87/85)R(85/86)R(86/87)=1+∈,

and use the e values as a control on the measured ratios. We note that

$$\begin{array}{l} \text{Average}\epsilon \approx 0\\ \text{Stanard deviation of}\;\epsilon \approx \{\Sigma (\text{relative s .d . of}\;R’s{)}^{2}{\}}^{1/2}. \end{array}$$

The relative standard deviations, computed from the residual standard deviations resulting from the linear fit of *R’s* in a cycle, can be used in the above expression.

A control chart on ϵ can then be constructed to monitor measurements within a run. If ϵ’s are predominately positive (negative), it is an indication of the presence of a systematic error at some, identifiable, point in the measurement procedure. Investigation as to its cause is in order.

The three-ratio scheme has been implemented in this laboratory (CBNM) for about 6 months now and seems to work well [2]. With computer controlled measurement and summary, the additional work is minimal once the software is prepared. A typical data summary sheet for the measurement of a silicon specimen is shown in Table 1, listing the three ratios measured in a cycle of ten scans, together with the ϵ’s calculated for each scan, extrapolated values at *t*_0_ and the relative standard deviations.

**Fig. 3:**
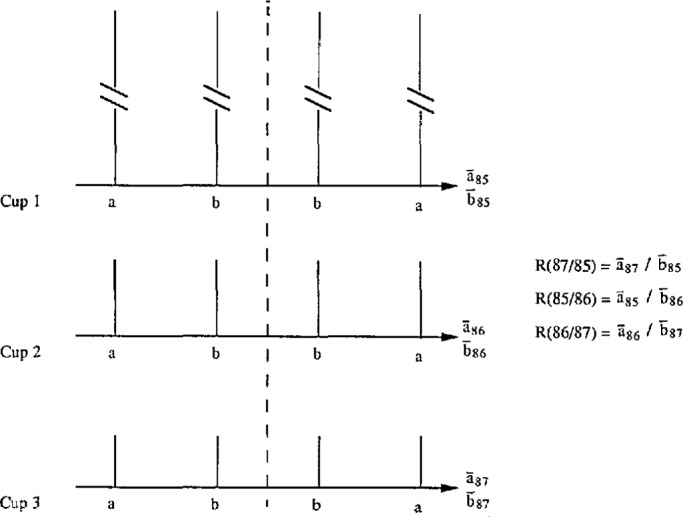
Scheme for three Faraday cups. One scan of a scheme using three Faraday cups is shown in this diagram. Two averages of ion currents of each ion beam, $\bar{a}$ and $\bar{b}$, are used to form the three ratios *R* (87/85), *R*(85/86), and *R* (86/87).

With the addition of the third ratio, there are also a number of features that can be used to check on the accuracy of the mass spectrometric measurements:

- We note that in Fig. 2, the three ratios can be computed by different pairing of the intensities, e.g.,
  $$\begin{array}{c} R\left(87/85\right)=I\left(2\right)/I\left(4\right),\;R\left(85/86\right)=I\left(1\right)/I\left(6\right),\\ R\left(86/87\right)=I\left(3\right)/I\left(5\right), \end{array}$$
  and so on. Ratios computed from different pairings can be compared. If they differ consistently, the cause should be investigated.
- If we denote 85 by A, 86 by B and 87 by C, then the sequence in Fig. 2 can be represented as
  ACBACBBCABCA.
  By permuting the positions of these isotopes, we could use also
  $$\begin{array}{l} \text{ABCABCCBACBA}\\ \text{BACBACCABCAB}\\ \text{BCABCAACBACB}\\ \text{CBACBAABCABC}\\ \text{CABCABBACBAC}. \end{array}$$
  The essential difference of these six sequences is the position of the major isotope A relating to the minor isotopes. If these six sequences yield ratios that are different beyond experimental errors, it is an indication that adjustments should be made on mass position, interference, or other factors.
- The ratio of the minor isotopes, *R* (86/87) is much more sensitive to small changes than the other two ratios where the major peak dominates the behavior of these ratios. For example, when a natural silicon sample is measured after an enriched ^29^Si, the mass spectrometer seems to remember the last measurement (by adsorption to the walls or other reasons) and yields a higher 86 intensity than actually present in the natural silicon. This effect shows more clearly in the ratio (86/87) than the other two. Hence it can be used to check whether there has been enough flushing and cleaning of the ion source to erase the memory.
- If we denote the three measured ratios by *R*_1_, *R*_2_, and *R*_3_=, then the least squares adjusted ratios are [5]:
  $$r_{1}={\left(\frac{{R_{1}}^{2}}{R_{2}R_{3}}\right)}^{1/3};\;r_{2}{\left(\frac{{R_{2}}^{2}}{R_{1}R_{3}}\right)}^{1/3};\;r_{3}{\left(\frac{{R_{3}}^{2}}{R_{1}R_{2}}\right)}^{1/3}.$$
  *r*_1_ and *r*_2_ can be used in Eq. (1). The relative standard deviations of the *r*’s is about 0.82 ( $\sqrt{2/3}$) of those for the *R* ’s.
- If a mass spectrometer has three Faraday cups to measure the three intensities separately (as is the case of the new spectrometer at CBNM), a similar scheme may be devised to yield three independent ratios as shown in Fig. 3. For each isotope, e.g., 85 using cup 1, four ion currents are measured at equal intervals in time in a scan. The two end ones are designated by *a*’s, and the two middle ones by *b’s.* Similarity, for 86 using cup 2, and 87 using cup 3, measured during the same time intervals as those of 85. Thus for each isotope, we have two averages, $\bar{a}$ and $\bar{b}$, both corresponding to time $\bar{t}$. Hence, three independent ratios can be computed, using the six averages ${\bar{a}}_{85}$, ${\bar{b}}_{85}$, ${\bar{a}}_{86}$, ${\bar{b}}_{86}$, ${\bar{a}}_{87}$, and ${\bar{b}}_{87}$, as follows:
  $$R\left(86/85\right)=\frac{{\bar{a}}_{86}}{{\bar{b}}_{85}};\;R\left(85/87\right)=\frac{{\bar{a}}_{85}}{{\bar{b}}_{87}};\;R\left(87/86\right)=\frac{{\bar{a}}_{87}}{{\bar{b}}_{86}},$$
  Here the *ϵ*’s calculated from the three independent ratios would also check the consistency of the three cups.

With the objective to determine the molar mass of silicon accurate to one part in 10^7^, thus requiring a precision in ratio measurements to parts in the 10^5^ range, it is imperative to investigate all avenues of improvements. The three-ratio scheme provides symmetry and redundancy and appears to be a helpful step in this direction.

In the above, we have used the three isotopes of silicon as an example to illustrate the three-ratio scheme. It is obvious that any three- or more- isotopes of a polyisotopic element can be treated in the same manner. The selection of the particular set of three isotopes is, of course, a decision the experimenter must make to suit his objectives.

## Figures and Tables

**Table 1:** Typical data summary sheet for a cycle of ten scans. Observed ratios of ion currents *R*(86/85), *R*(85/87) and *R* (87/86) arc listed in columns 2, 3, and 4, respectively, for the ten scans. The (1 + *ϵ*) values area calculated for each scan and for the extrapolated values at *t*_0_

Scan no.	*I*(^29^Si)/*I*(^28^Si)	*I*(^28^Si)/*I*(^30^Si)	*I*(^30^Si)/*I*(^29^Si)	I + *ϵ*
1	0.050 597 5	29.880 0	0.661 443	1.000 005
2	0.050 597 1	29.871 2	0.661 547	0.999 860
3	0.050 606 0	29.864 9	0.661 637	0.999 961
4	0.050 615 4	29.858 5	0.661 706	1.000 036
5	0.050 617 5	29.849 7	0.661 798	0.999 922
6	0.050 622 9	29.842 2	0.661 888	0.999 913
7	0.050 628 5	29.834 4	0.661 954	0.999 862
8	0.050 636 2	29.826 7	0.662 056	0.999 910
9	0.050 642 7	29.819 4	0.662 123	0.999 895
10	0.050 645 7	29.812 9	0.662 238	0.999 910

Mean				0.999 927
Std. dev.				57

		Extrapolation		

Value (*t*=*t*_0_)	0.050 574 5	29.906 7	0.661 149	0.999 998
Std. dev.	20	7	11	48

Rel. std. dev.	3.9·10^−5^	2.3·10^−5^	1.7·10^−5^	4.8.10^−5^

## Footnotes

1 The molar mass of a particular specimen of silicon thus determined was used in a project, in collaboration with the Physikalisch-Technische Bundesanstalt (PTB), Braunschweig, Germany, for the redetermination of the Avogadro Constant [1,2].
